# Upcycling of Fe-bearing sludge: preparation of erdite-bearing particles for treating pharmaceutical manufacture wastewater

**DOI:** 10.1038/s41598-020-70080-4

**Published:** 2020-08-03

**Authors:** Tongke Hu, Huaimin Wang, Ruyan Ning, Xueling Qiao, Yanwen Liu, Wenqing Dong, Suiyi Zhu

**Affiliations:** Science and Technology Innovation Centre for Municipal Wastewater Treatment and Water Quality Protection, Northeast Normal University, Changchun, 130117 China

**Keywords:** Environmental sciences, Environmental chemistry, Hydrology

## Abstract

Groundwater treatment sludge is a type of solid waste with 9.0–28.9% wt.% Fe content and is precipitated in large quantity from backwash wastewater in groundwater treatment. The sludge is mainly composed of fine particles containing Fe, Si and Al oxides, such as ferrihydrite, quartz and boehmite. The Fe oxides mostly originate from the oxidation of ferrous Fe in groundwater, whilst the silicate/aluminium compounds mainly originate from the broken quartz sand filter in the backwash step. In general, the sludge is firstly coagulated, dewatered by filter pressing and finally undergoes harmless solidification before it is sent to landfills. However, this process is costly (approximately US$66.1/t) and complicated. In this study, groundwater treatment sludge was effectively recycled to prepare novel erdite-bearing particles via a one-step hydrothermal method by adding only Na_2_S·9H_2_O. After hydrothermal treatment, the quartz and boehmite of the sludge were dissolved and recrystallised to sodalite, whilst ferrihydrite was converted to an erdite nanorod at 160 °C and a hematite at 240 °C. SP160 was prepared as fine nanorod particles with 200 nm diameter and 2–5 μm length at a hydrothermal temperature of 160 °C. Nearly 100% OTC and its derivatives in pharmaceutical manufacture wastewater were removed by adding 0.1 g SP160. The major mechanism for the removal was the spontaneous hydrolysis of erdite in SP160 to generate Fe oxyhydroxide and use many hydroxyl groups for coordinating OTC and its derivatives. This study presents a novel method for the resource reutilisation of waste groundwater treatment sludge and reports efficient erdite-bearing particles for pharmaceutical manufacture wastewater treatment.

## Introduction

Fe-bearing sludge is common in the metallurgical and chemical industries^1–3^. Sludge contains 6.2–33.6% Fe, in the form of ferrihydrite, hematite, magnetite and andradite, and impurity minerals, such as quartz, muscovite, albite and boehmite^1,4^. In China, tons of sludge are dumped and uncovered in massive piles^2^. When rain occurs, Fe may leach and contaminate the soil and nearby surface water^5^. Through strict environmental regulations, sludge is commonly dewatered and solidified before it is transferred to a safety landfill, but this process is costly and complicated^2,6–8^.

Resource reutilisation of sludge is an alternative strategy to reduce pollution and disposal cost. Many approaches have been developed to recycle sludge as a building material^9^, catalyst^10^ and adsorbent^3,4,6,11^. Amongst these approaches, the conversion of sludge to adsorbent is the most effective and simple, and the produced adsorbents are widely used in the adsorption of heavy metals^4,6,12^ and cationic organics^1,3,11,13,14^. For instance, groundwater treatment sludge and flocculent iron mud are rich in ferrihydrite and are directly applied to adsorb heavy metals, such as Cu and Zn^15^. Ferrihydrite is effectively converted to maghemite and magnetite via hydrothermal route or calcination with the addition of a reductant, such as glycol^11^ and ascorbic acid^4^. Thus, the resulting product has good separation performance and can be magnetically collected after adsorption. Although the product has abundant surface functional groups (≡Me–OH,Me represents Fe, Al and Si) to adsorb contaminants, the conversion of weakly crystallised ferrihydrite to well-formed maghemite and magnetite leads to a small coordination number of surface functional groups and, consequently, low adsorption performance^3,4^. When approximately 18.9% ferrihydrite in the precipitated sludge of a groundwater plant was converted to maghemite and hematite, the total surface site concentration of the resulting product decreased from 1.03 mmol/g to 0.51 mmol/g^3^. Thus, a new, efficient method that improves the adsorption performance of a prepared product must be developed.

Amongst Fe-bearing minerals, erdite is a special 1-D nanomaterial comprising (FeS_2_)_*n*_^*n-*^^16^. It is metastable in neutral and mildly acidic solutions and is easily decomposed into Fe oxyhydroxide and S-containing compounds^17^. The new Fe oxyhydroxide has numerous surface hydroxyl groups, similar to the hydrolysed product of a polyferric sulphate flocculant, and exhibits high adsorption capacity for arsenic^18^, rare-earth metals^19^ and phosphate^20^. However, the conversion of Fe oxides in sludge to erdite and the application of erdite-bearing materials in wastewater treatment have not been reported.

In this study, Fe-bearing sludge was sampled in a groundwater plant and hydrothermally converted to erdite-bearing particles by adding only Na_2_S·9H_2_O. The conversion mechanism of Fe-bearing minerals in the sludge to erdite was investigated. Also, oxytetracycline (OTC) is a typical antibiotic common in pharmaceutical manufacture wastewater. Hence, it was targeted to determine the adsorption performance of the prepared particles (SPs). The application of prepared erdite-bearing particles in the treatment of OTC-bearing pharmaceutical manufacture wastewater was also explored.

## Results and discussion

### SP formation

**Figure 1:**
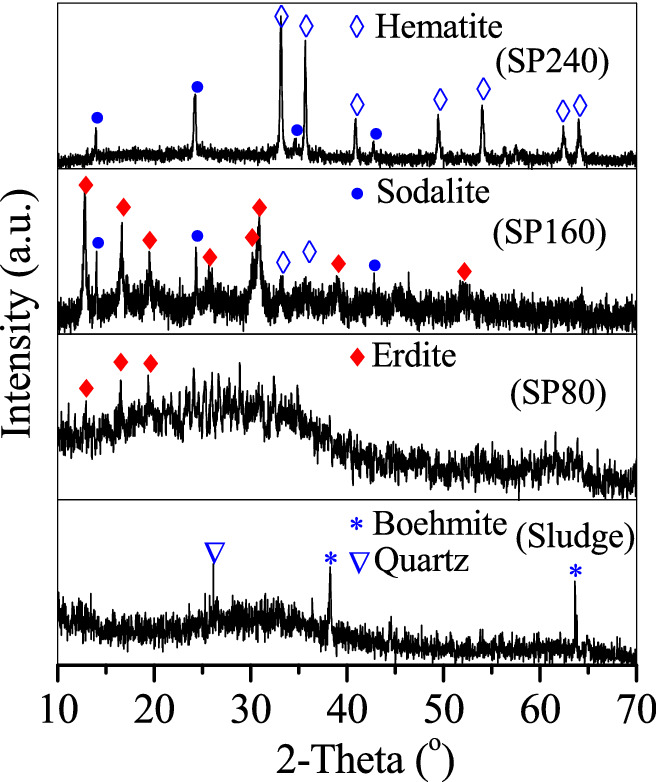
XRD curves of the sludge, SP80, SP160 and SP240.

Figure 1 shows the x-ray diffractometry (XRD) patterns used for the analysis of sludge and SP composition. The typical peaks of quartz and boehmite were recorded at the curve of the sludge. The sludge XRD pattern showed a typical peak at 2*θ* = 26.1° that belongs to quartz (JCPDS 46–1,045)^21,22^ and two peaks at 2*θ* = 38.2° and 63.6° that belong to boehmite (JCPDS 74–1895)^23^. Sludge impurities were also rechecked, and a valuable reference of the sludge composition^24^ was cited. Quartz and boehmite, which were primarily obtained from the broken quartz sand media and hydrolysed coagulant (e.g. polymeric aluminium), were the major impurities in the sludge. However, no peaks of Fe oxides were observed, demonstrating that Fe oxides in the sludge were weakly crystallised. Fe oxides were mainly obtained from the oxidation of Fe^2+^ in groundwater to Fe^3+^ in hydrolysed form. The hydrolysed product of Fe^3+^ in the sludge was further identified to be ferrihydrite through the Mossbauer experiment (Fig. S1). After the diffraction area of Fe oxides in the sludge was calculated, the relative area percentage of ferrihydrite was close to 100% (Table S1), suggesting the abundance of ferrihydrite in the sludge.

After hydrothermal treatment at 80 °C with additional Na_2_S·9H_2_O, no quartz and boehmite peaks in the curves of SP80 were observed, but three weak peaks at 2*θ* = 12.8°, 16.6° and 19.5° appeared in accordance with the erdite formation. When the hydrothermal temperature was 160 °C, SP160 showed intensified erdite peaks, hematite peaks at 2*θ* = 33.2° and 35.7° and sodalite peaks at 2*θ* = 14° and 24.3°. However, at the hydrothermal temperature of 240 °C, the formed SP240 showed that the erdite peaks disappeared, whereas the sodalite and hematite peaks were intensified. Hence, with the addition of Na_2_S·9H_2_O, quartz and boehmite were dissolved at 80 °C and recrystallised to sodalite at 160 °C and 240 °C, respectively. Accordingly, ferrihydrite in the sludge was transformed to erdite at 80 °C and apparently at 160 °C and to hematite completely at 240 °C.

**Figure 2:**
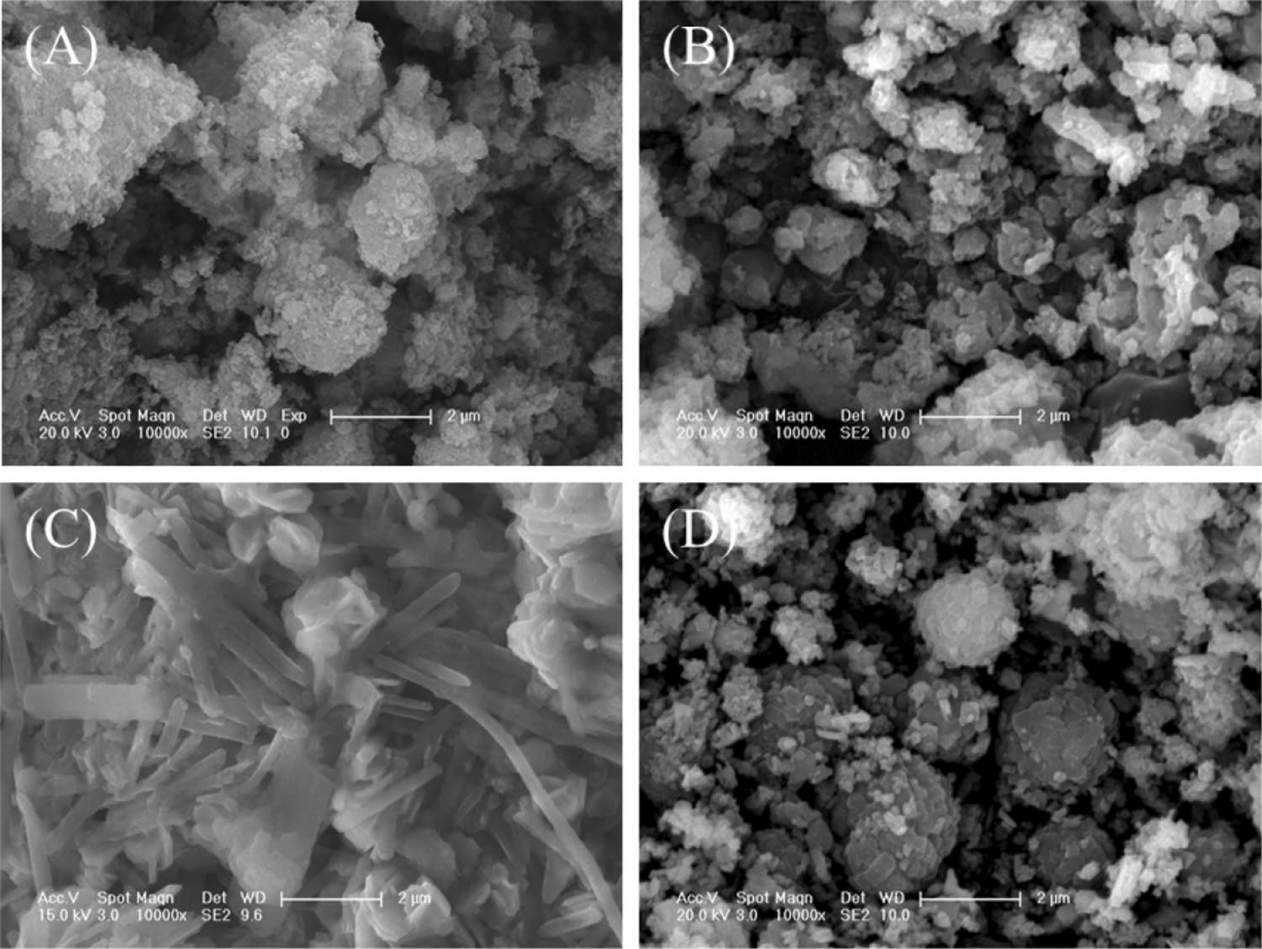
SEM pictures of (**A**) the sludge, (**B**) SP80, (**C**) SP160 and (**D**) SP240.

The sludge comprised irregular aggregates (Fig. 2A). After hydrothermal treatment, the product SP80 showed a morphology similar to that of the sludge (Fig. 2B). In comparison with SP80, SP160 showed nanorod particles with a diameter of 200 nm and a length of 2–5 μm (Fig. 2C) belonging to erdite (Fig. 1 SP160). However, SP240 showed that the nanorod particles disappeared and that spherical particles with a diameter of 2 μm (Fig. 2D) belonged to sodalite (Fig. 1 SP240).

**Figure 3:**
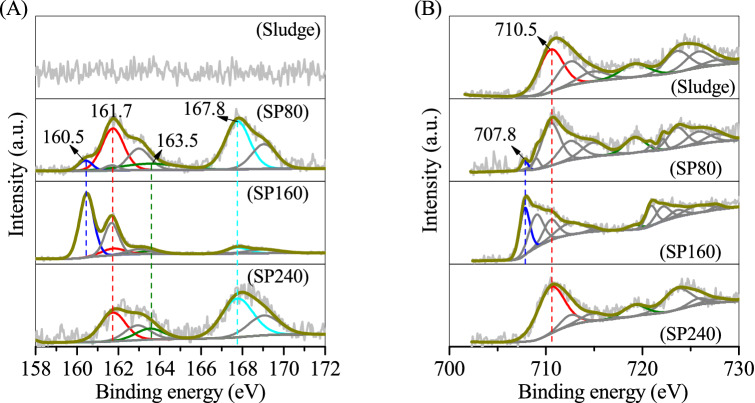
XPS curves of (**A**) S 2*p* and (**B**) Fe 2*p* on the sludge and prepared particles.

The valence states of S and Fe on the sludge and SPs were determined using an X-ray photoelectron spectrometer (XPS). Figure 3A shows that no evident S 2*p*3/2 peak was recorded in the curve of the sludge because of the absence of S. Through the addition of Na_2_S·9H_2_O, four major S 2*p*3/2 peaks at binding energies of 160.5, 161.7, 163.5 and 167.8 eV were observed in SP80, corresponding to the structural S in the (FeS_2_)_*n*_^*n*-^ chain of erdite, S^2−^, polysulfide and SO_4_^2−^, respectively. The peak of structural S in erdite was intensified in SP160, consistent with the well-crystallised erdite in SP160 (Figs. 1 SP160 and 2C). However, it disappeared in SP240 owing to the absence of erdite in SP240 (Fig. 1 SP240). For the Fe 2*p* spectra (Fig. 3B), the sludge exhibited a major peak at the binding energy of 710.5 eV that belongs to Fe^3+^ in the Fe–O structure of ferrihydrite in the sludge. After hydrothermal treatment, a weak peak at a binding energy of 707.8 eV was observed in SP80, corresponding to the structural Fe in the polymeric chain of (FeS_2_)_*n*_^*n*-^, similar to that in chalcopyrite^25^. The peak of structural Fe in erdite was sharp in SP160 but disappeared in SP240. This phenomenon is consistent with the conversion of ferrihydrite to erdite at 160 °C and to hematite at 240 °C.

### Conversion mechanism of the sludge to SPs

**Figure 4:**
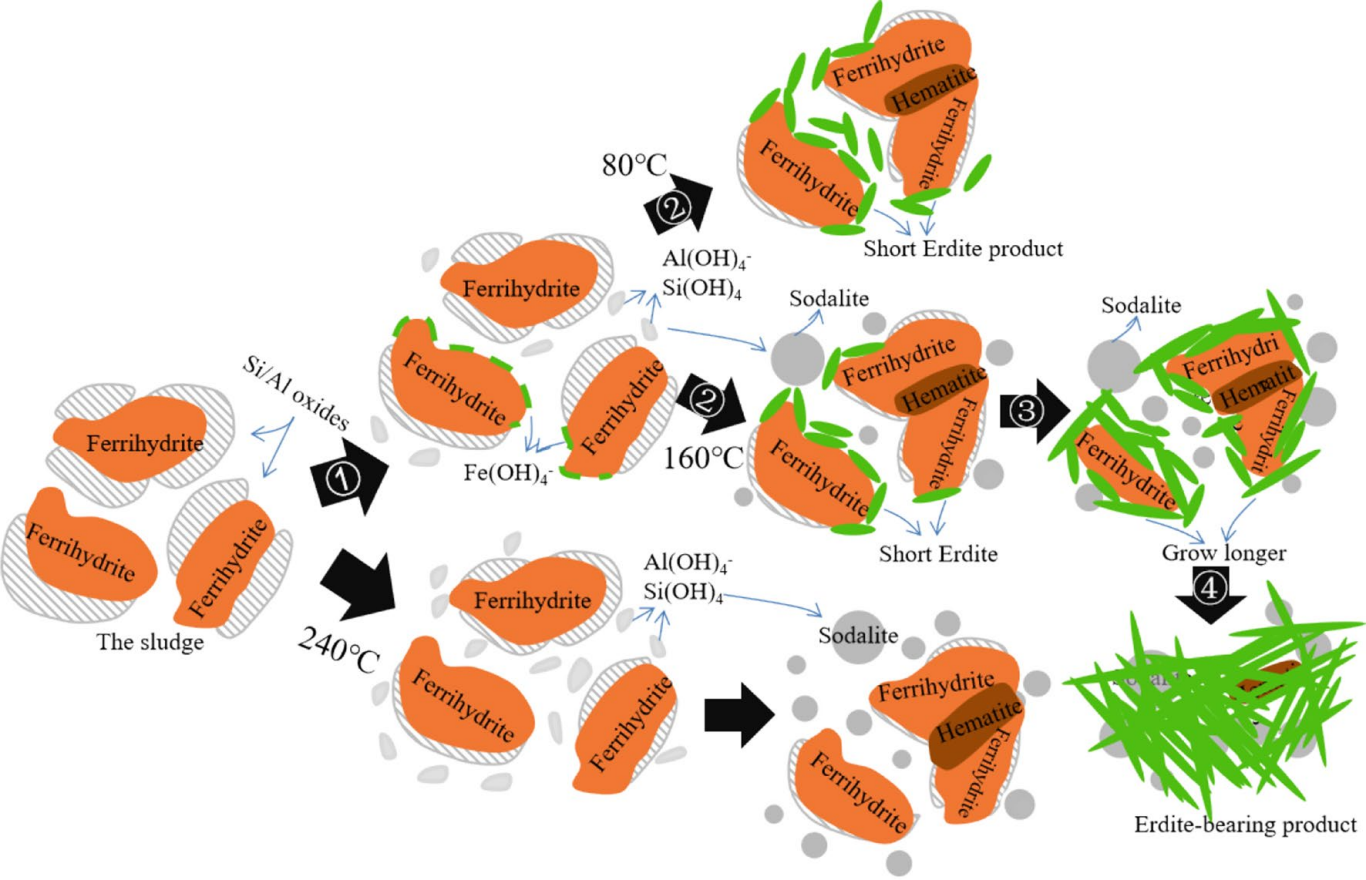
Illustration of sludge conversion to SPs.

When Na_2_S·9H_2_O was introduced in the hydrothermal system, it was rapidly hydrolysed to HS^−^ and OH^−^, leading to a solution pH above 13.6. Thus, quartz and boehmite were dissolved to generate Al(OH)_4_^-^ and Si(OH)_4_ (Fig. 4, Step 1). This dissolution agreed with the high concentration of Al/Si in the supernatant (Fig. S2). Subsequently, the nucleation and growth of Si/Al compounds occurred^26^ slowly at 80 °C and rapidly at 160 °C to generate a stable phase, such as sodalite (Fig. 4, Step 2). Next, the nucleation and growth of Si/Al compounds steadily accelerated as the temperature increased to 240 °C.

The ferrihydrite in the sludge had many surface hydroxyl groups for adsorbing Si/Al oxides. The Si/Al oxides were hydrothermally dissolved in the presence of Na_2_S to regenerate the surface hydroxyl groups. Thus, Fe on the ferrihydrite surface was uncovered and then attacked by OH^−^ under a strong alkaline condition to generate Fe(OH)_4_^-^ (Eq. (1); Fig. 4, Step 1) in the solution. This phenomenon agreed with the Fe concentration of 5–15 mg/L in the supernatant after hydrothermal reaction (Fig. S2). Fe(OH)_4_^-^ was the sole stable Fe hydroxide^27^, and it reacted preferentially with the HS^−^ group to release a hydroxyl group via replacement reaction (Eq. (2)) instead of undergoing a redox reaction between the structural Fe in Fe(OH)_4_^-^ and the S in HS^−^^28^. Thus, Fe(OH)_3_HS^−^ was generated. The polymerisation of two adjacent Fe(OH)_3_HS^−^ spontaneously occurred (Fig. 4, Step 2) via dewatering reaction (Eq. (3)). In the presence of adequate Fe(OH)_4_^-^, the polymerisation reaction continued, thus forming a polymer (FeS_2_)_*n*_^*n*-^ chain as the final product (Fig. 4, Steps 3 and 4). The dewatering reaction between the Fe(OH)_3_HS^-^ groups accelerated with the increase of hydrothermal temperature from 80 °C to 160 °C, thereby promoting the formation of well-crystallised erdite in SP160.

1 $$Fe(OH{)}_{3}+O{H}^{-}\to {Fe(OH)}_{4}^{-}$$

2 $${Fe(OH)}_{4}^{-}+{HS}^{-}\to Fe(OH{)}_{3}H{S}^{-}+{OH}^{-}$$

3 $$2Fe(OH{)}_{3}H{S}^{-}\to 2{H}_{2}O+({Fe}_{2}{S}_{2}{(OH)}_{4}{)}^{2-}$$

During the hydrothermal process, the conjunction of adjacent hydroxyl groups on each ferrihydrite also occurred to generate a Fe–O–Fe bond. This conjunction was irreversible with hematite as the final product but was inhibited when the impurities Si/Al oxides (e.g. quartz and boehmite) were adsorbed on the ferrihydrite surface^29,30^. When the hydrothermal temperature was 240 °C, the dissolution of Si/Al oxides was accelerated to regenerate free hydroxyl groups on the ferrihydrite surface. In the presence of adequate free hydroxyl groups, the conjunction reaction occurred prior to the chemical reaction between the free OH^−^ and the surface Fe of ferrihydrite, thus inhibiting Fe(OH)_4_^-^ generation. Subsequently, the following reactions of erdite formation were completely stopped.

### Effective adsorption of OTC on the hydrolysed particle surface

The distribution coefficient (*k*_d_) was a useful index for comparing the adsorption capacities of raw sludge and prepared SPs under the same experimental conditions^31,32^, and calculated in the following equation.

4 $$ k_{d} = \frac{{q_{e} }}{{C_{e} }}, $$

In the equation, *q*_e_ is the amount of oxytetracycline (OTC) on the raw sludge and prepared SPs after adsorption, whilst *C*_e_ is the equilibrium concentration of OTC in the solution.

**Figure 5:**
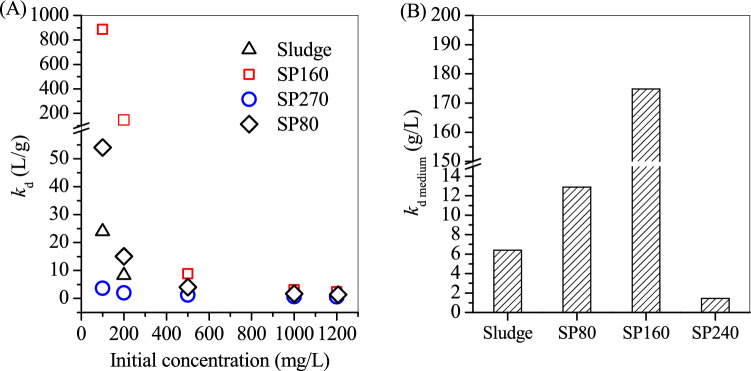
(**A**) *k*_d_ and (**B**) *k*_d medium_ values of oxytetracycline on the sludge, SP80, SP160 and SP240.

Figure 5 showed that with the increase of the initial concentration of OTC, the corresponding *k*_d_ also decreased. In general, the surface sites of sludge and SPs became saturated with OTC when its initial concentration was at a high level^32^, e.g. 1,000 mg/L, led to large number of free OTC remaining in the solution, resulting in a low *k*_d_. But at a low initial concentration of OTC (e.g. 100 mg/L), SP160 exhibited a high value of *k*_d_ in comparison with the raw sludge, SP80 and SP240, suggesting that SP160 had desirable performance for adsorption of OTC. Accordingly, by adding SP160, OTC was residual at a low level (0.56 mg/L) at the initial concentration of OTC of 100 mg/L, apparently lower than those of sludge (8.5 mg/L), SP80 (7.8 mg/L) and SP240 (57.6 mg/L). The plots of *k*_d_ versus initial concentration did not yield a straight line (Fig. 5A). Thus, an average *k*_d_ value (*k*_d medium_) was calculated in accordance with Lu and Xu’s method^33^, and used to compare the adsorption performance of sludge and SPs for OTC adsorption. Figure 5B showed that the calculated *k*_d medium_ of sludge and SPs were in the following order: SP160 > SP80 > sludge > SP240, in agreement with the order of maximum adsorption capacities of sludge and SPs. SP240 showed a very low *k*_d medium_, indicating that its fairly ineffective for OTC adsorption.

In the experiment, the product SP160, which was synthesised at 160 °C, was spontaneously hydrolysed in electroplating wastewater treatment and exhibited a superior adsorption capacity of OTC compared with products SP80 and SP240. Thus, SP160 was regenerated in the following experiment.

**Figure 6:**
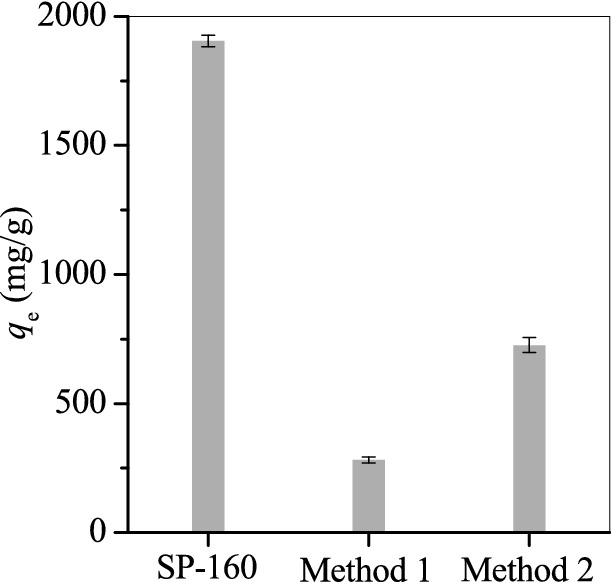
Reuse of the precipitate of hydrolysed SP160 for oxytetracycline adsorption.

The used SP160 was regenerated with 15% NaCl solution at pH 5 for 48 h (method 1) or by calcination at 450 °C for 5 h (method 2). The results showed that SP160 was regenerated easily by using NaCl solution as the desorption agent. However, the adsorption capacity of OTC dramatically decreased from 1,905.2 mg/g to 281.5 mg/g (Fig. 6). After calcination at 450 °C, the regenerated SP160 also showed a low adsorption capacity of 726.4 mg/g. This result indicated that the reusability of SP160 was not feasible.

### Adsorption mechanism

**Figure 7:**
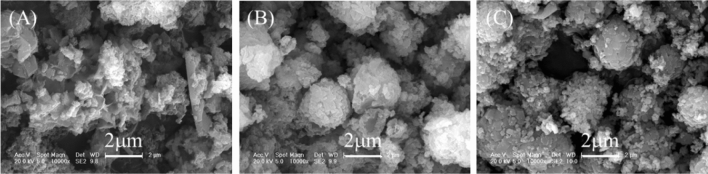
SEM pictures of (**A**) SP80, (**B**) SP160 and (**C**) SP240 after oxytetracycline adsorption.

**Figure 8:**
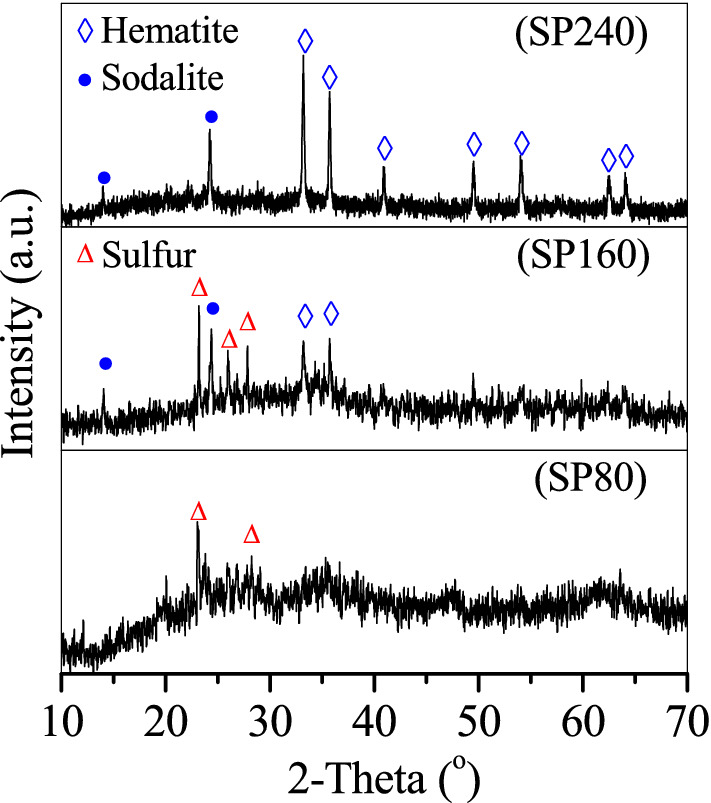
XRD patterns of the prepared particles after oxytetracycline adsorption.

After OTC adsorption, SPs were collected and then characterised via XRD, scanning electron microscope (SEM) and XPS. The results indicated that the morphology of SP80 did not change clearly (Fig. 7A). However, its XRD patterns showed that the erdite peak disappeared and new peaks of sulphur appeared (Fig. 8 SP80) instead. The formation of these new peaks could be attributed to the hydrolysis of erdite in SP80 to generate S. For SP160, the nanorod erdite was transformed to irregular blocks after OTC adsorption (Fig. 7B). Subsequently, strong peaks of erdite were replaced by new peaks of sulphur (Fig. 8 SP160), similar to the erdite hydrolysis in SP80. Moreover, indicative peaks of hematite and sodalite were recorded in the curve of SP160. Thus, the two minerals were stable during OTC adsorption. In comparison with the morphology and XRD patterns of SP160, those of SP240 were almost unchanged (Figs. 7C and 8 SP240). Hence, OTC adsorption mainly occurred on the SP240 surface.

**Figure 9:**
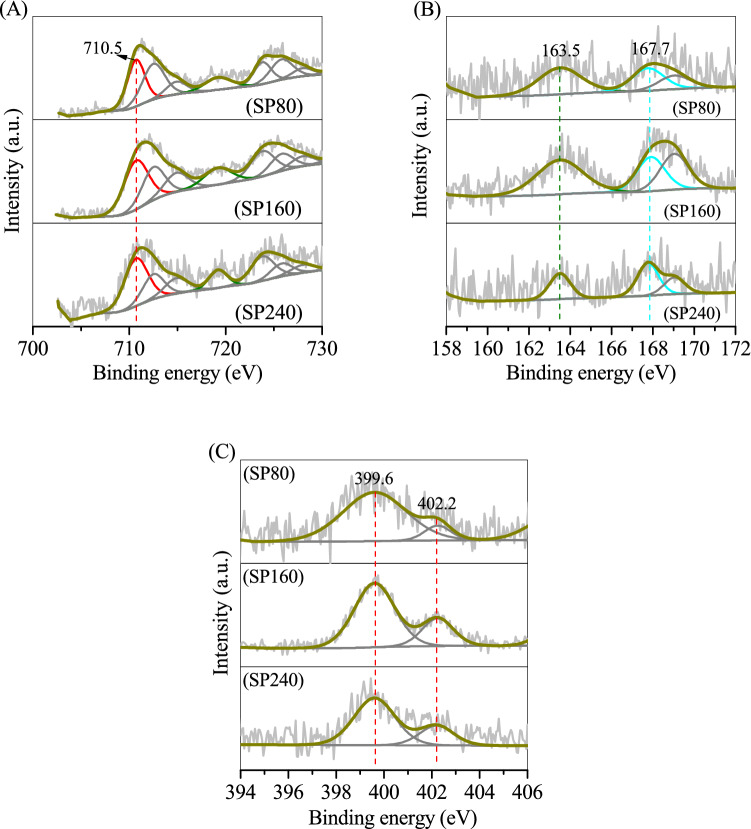
XPS curves of (**A**) Fe2*p*, (**B**) S2*p* and (**C**) N1*s* on the prepared particles after oxytetracycline adsorption.

The SPs after OTC adsorption were also characterised via XPS. The SP80 and SP160 spectra showed that the typical peak of Fe 2p3/2 shifted to a high binding energy of 710.5 eV (Fig. 9A SP80 and SP160), corresponding to the Fe–O bond in Fe oxyhydroxide from the hydrolysed erdite. Sulphur and sulphate peaks were also recorded in the curves of SP80 and SP160, whilst the typical peak of structural S in the S–Fe bond of erdite was eliminated (Fig. 9B SP80 and SP160). Thus, the hydrolysis of erdite generated a mixture of Fe oxyhydroxide and sulphur. For SP240, the spectra of Fe and S were almost unchanged (Figs. 9A SP240 and 9B SP240), indicating the stability of SP240 during OTC adsorption. The N 1 s XPS (Fig. 9C) spectrum shows that the two binding energies of 399.6 and 402.2 eV could be attributed respectively to the C–N = C bond of OTC and –NH_3_^+^ on the side chain of OTC^34^, demonstrating the adsorption of OTC on SPs. OTC is an amphiprotic compound,its cation is below pH 3.3, the zwitterion is in the pH range of 3.3–7.7, and the anion is at pH > 7.3^35^. During adsorption, the solution pH increased slightly from 5 to 6.8, causing the zwitterionic OTC to come close easily to the SP surface and react with the surface hydroxyl groups to form an OTC-complexed compound.

Amongst the three SPs, SP160, which was a mixture of erdite and sodalite, exhibited the optimal OTC adsorption capacity. When it was dispersed in the OTC-bearing solution, erdite was spontaneously hydrolysed via the reverse reaction (Eqs. (1)–(3)) to generate Fe oxyhydroxide and release HS^−^ to the supernatant. In the presence of enough dissolved oxygen, redox reaction between HS^−^ and dissolved oxygen occurred with the generation of sulphur^17^. The newly formed Fe oxyhydroxide had a special structure in which one Fe atom had an average coordination number of hydroxyl group of 5.4, which is higher than that of aggregated ferrihydrite and hematite^36^. Thus, the hydrolysed SP160 used enough hydroxyl groups for OTC coordination. In addition, sodalite in SP160 had surface functional groups, such as ≡Si–OH and ≡Al–OH^37^, for OTC adsorption. However, it contributed little to OTC removal compared with Fe oxyhydroxide. Only a small portion of erdite was generated in SP80, leading to a lower OTC adsorption capacity of SP80 compared with that of SP160. Conversely, for SP240, most of the ferrihydrite was converted to hematite, causing the SP240 agglomeration and, subsequently, the lowest OTC adsorption.

### Pharmaceutical manufacture wastewater treatment

**Figure 10:**
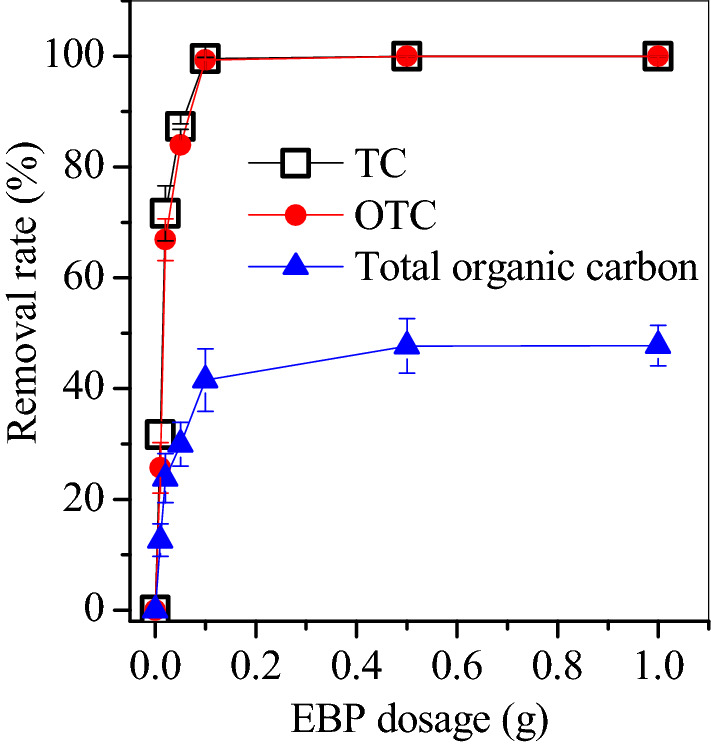
Removal rates of tetracycline, oxytetracycline and total organic carbon in pharmaceutical manufacture wastewater.

Given that SP160 had the optimal OTC adsorption capacity, it was used in the treatment of OTC-containing pharmaceutical manufacture wastewater. pharmaceutical manufacture wastewater in this study contained 27.2 mg/L tetracycline (TC), 113.5 mg/L OTC and 483.6 mg/L total organic carbon (TOC). The addition of 0.1 g SP160 removed nearly 100% OTC and TC, whereas the TOC removal was below 50% even if the amount of SP160 was 1 g (Fig. 10). Thus, SP160 was efficient in the removal of TC and OTC and could be a desirable adsorbent in the pre-treatment of pharmaceutical manufacture wastewater.

### Potential application

The hydrothermal method makes the conversion of ferrihydrite in the sludge to erdite easy and feasible. Common impurities, such as Si/Al oxides, were not involved in the erdite formation. Thus, the method could be developed to recycle other ferrihydrite-bearing solid waste, such as cold rolling iron mud and flocculent sludge to prepare erdite. The prepared erdite was metastable in neutral and weakly acidic solutions, and it spontaneously hydrolysed to Fe oxyhydroxide. The new Fe oxyhydroxide had good adsorption performance for OTC and its derivatives. Thus, the erdite-bearing particles could be applied to pharmaceutical manufacture wastewater treatment. Ferrihydrite was also converted to erdite at 80 °C. Therefore, such erdite-bearing particles could be synthesised at normal temperature, possibly decreasing the total cost of erdite synthesis to a great extent.

## Materials and methods

### Fe-bearing sludge collection

Fe-bearing sludge was collected at the clarifier bottom of the Kulunqi groundwater plant (Inner Mongolia, China) and dried at 110 °C overnight. The composition of dried sludge was analysed using an X-ray fluorescence spectrometer (S4-Explorer, Bruker, Germany) and was found to consist of 23.2% Fe, 7.5% Al and 3.7% Si.

### Conversion of the sludge to SPs

The sludge was hydrothermally converted to SPs by mixing 2.9 g Na_2_S·9H_2_O and 1 g sludge in 30 mL of deionised water, followed by dumping into a 50 mL Teflon vessel. The vessel was sealed and placed in a drying oven (101-3S, Shanghai-Licheng, China) and then heated at 80 °C for 10.5 h. Blackish particles (denoted as SP80) were collected at the vessel bottom and then freeze-dried at − 80 °C overnight. Control experiments were also performed by varying the heating temperature from 80 °C to 160 °C and 240 °C, and the product particles were named as SP160 and SP240, respectively.

### OTC adsorption and wastewater treatment

OTC is a common pollutant in pharmaceutical manufacture wastewater^38^. It was used to evaluate the adsorption capacity of SPs using the following steps. A stock solution with an OTC concentration ranging from 0.5 to 2,000 was adjusted to pH 5 by using 5% HCl or 5% NaOH. The stock solution (100 mL) and SPs (0.02 g) were mixed in a 250 mL beaker under stirring at 80 rpm for 2 h at room temperature. The added SPs were collected by centrifugation at 6,000 rpm for 5 min. The OTC concentration was determined using a high-performance liquid chromatograph (Waters-2695, Waters Alliance, USA), with a C18 column (ODS–C18, 46 mm × 255 mm, Waters Alliance, USA), followed by UV detection at 360 nm. A mixture of 0.01 M oxalic acid–acetonitrile (80:20, *v*/*v*) was used as mobile phase with a flow rate of 1 mL/min. The retention time was 5.25 min, and the detection limit was 0.01 mg/L.

Adsorbent regeneration was carried out by treating with 15% NaCl solution at pH 5 for 48 h (method 1) or by calcination at 450 °C for 5 h (method 2). The adsorbent after regeneration treatment (0.02 g) was put into 100 mL oxytetracycline with a dose of 1,000 mg/L and a equilibrium time of 2 h.

SP160 exhibited the optimal OTC adsorption capacity and was applied to the treatment of OTC-containing pharmaceutical manufacture wastewater. Wastewater sample was obtained from the effluent of the purification workshop of the Huawei Pharmacy Company (Yushu, China). Approximately 0.01 g of SP160 was added to 50 mL of wastewater in a 100 mL conical flask following the above mentioned procedure. In the experiments, the dosage of SP160 was also increased from 0.01 to 0.02, 0.05, 0.1, 0.2, 0.5 and 1 g, separately, and the concentrations of OTC and TOC in the treated wastewater were determined. The concentration of TOC was measured using a high-temperature TOC analyser (TOC-5000A, Shimadzu, Japan) with an ASI-5000 auto sampler according to the method of APHA^39^.

### Characterisation

The sludge and the SPs were characterised using an X-ray diffractometer (Rigaku, Rint2200, Japan), an XPS (ADES 400, VG Scientific, UK) and a SEM (JSM-6400, Jeol, Japan).

## Conclusion

The sludge in this study was a mixture of ferrihydrite, quartz and boehmite. The impurities, quartz and boehmite, were hydrothermally dissolved in the presence of Na_2_S·9H_2_O and then recrystallised to generate sodalite, whilst ferrihydrite was converted to an erdite nanorod at 160 °C and hematite aggregates at 240 °C. The erdite-bearing product, SP160, was synthesised after hydrothermal treatment at 160 °C. SP160 was found to be effective in the removal of OTC and its derivatives from pharmaceutical manufacture wastewater. Thus, SP160 can serve as an effective adsorbent in the treatment of pharmaceutical manufacture wastewater.

## Supplementary information

Supplementary file1
