# Supplementary material for: Upcycling of Fe-bearing sludge: preparation of erdite-bearing particles for treating pharmaceutical manufacture wastewater
Source: Sci Rep. 2020 Aug 3;10:12999. doi: 10.1038/s41598-020-70080-4 (PMC7400646; doi:10.1038/s41598-020-70080-4)
Supplement: Supplementary file 1 — Supplementary file1 [file 41598_2020_70080_MOESM1_ESM.docx]

**Upcycling of Fe-bearing sludge: preparation of erdite-bearing particles for treating pharmaceutical manufacture wastewater**

Tongke Hu ^a^, Huaimin Wang ^a^, Ruyan Ning ^a^, Xueling Qiao ^a^, Yanwen Liu ^a^, Wenqing Dong ^a^, Suiyi Zhu ^a,*^

**Figure S1** Mössbauer spectra of the sludge.

**Table S1** Mössbauer parameters of the sludge.

| Sample | Component | IS (mm/s) | QS (mm/s) | H_in_ (KOe) | HWHM (mm/s) | Area (%) |
| --- | --- | --- | --- | --- | --- | --- |
| The sludge | Ferrihydrite | 0.27±0 | 0.85±0 |  | 0.27±0 | 100% |

Note: IS: the isomer shift; QS: is the quadruple split; H_in_: the hyperfine field; HWHM: the half width at half maximum; Area: the relative absorption area.

**Figure S2** the concentrations of Al, Si, and Fe in the supernatant after hydrothermal treatment.

The product SP160, which was synthesised at 160 °C, showed the optimal adsorption capacity of OTC amongst the two other synthesised particles, SP80 and SP240. Subsequently, the adsorption of OTC on SP160 was simulated using pseudo-first- (Eq. 1) and pseudo-second-order models (Eq. 2) (Fig. S3) separately. The two kinetic models were expressed as follows:

$q_{t}=q_{e}(1-exp(-k_{1}t)$), (1)

$q_{t}=\frac{k_{2}q_{e}^{2}t}{1+k_{2}q_{e}t}$, (2)

where *q_e_* and *q_t_* are the adsorption capacities (mg/g) of OTC at equilibrium and at any time *t*, respectively; *k_1_* is the pseudo-first-order model rate constant (1/min); and *k_2_* is the pseudo-second-order adsorption rate constant (×10^−3^ g/mg.h).

The kinetic parameters are summarised in Table S2. The adsorption of OTC on SP160 was equilibrium in 1 h, and its rate was higher than that of other carbon materials, such as corn stover-derived biochar ^1^ and cotton-made activated carbon ^2^. The adsorption data fitted well with the pseudo-second-order model with a high correlation coefficient (*R^2^*) of 0.997, suggesting that the adsorption of OTC based on the chemisorption process was a rating controlling step ^2, 3^. The adsorption of OTC by other adsorbents, such as zeolite 13X ^3^, hydroxyapatite ^4^ and activated carbon ^1, 2^, was also observed following the pseudo-second-order model.

**Figure S3** Adsorption kinetics of oxytetracycline on SP160

**Table S2** Parameters and regression coefficients (*R^2^*) of the kinetic models.

| Kinetic model | Parameters | SP160 |
| --- | --- | --- |
| Pseudo-first-  Order | *q*_e,exp_ (mg/g) | 1821.1 |
|  | *k_1_* (1/min) | 3.545 |
|  | *R^2^* | 0.805 |
| Pseudo-second-  order | *q*_e,cal_ (mg/g) | 1980.2 |
|  | *k_2_* (×10^−3^ g/mg.h) | 5.817 |
|  | *R^2^* | 0.997 |

1. Zhang, M. et al. Corn stover–derived biochar for efficient adsorption of oxytetracycline from wastewater*.* *Journal of Materials Research*. **34**, 3050-3060 (2019).

2. Sun, Y. et al. Preparation of activated carbon derived from cotton linter fibers by fused NaOH activation and its application for oxytetracycline (OTC) adsorption*.* *Journal of Colloid and Interface Science*. **368**, 521-527 (2012).

3. Li, Y., C. Zhao, and H. Deng. Oxytetracycline removal in aqueous by two kinds of zeolites with different bore diameter*.* *Huan jing ke xue= Huanjing kexue*. **31**, 990-995 (2010).

4. Harja, M. and G. Ciobanu. Studies on adsorption of oxytetracycline from aqueous solutions onto hydroxyapatite*.* *Science of The Total Environment.* **628-629**, 36-43 (2018).
